# Gangrenous Cholecystitis in a 54-Year-Old Male With Schizophrenia and Other Barriers to Care: A Case Report

**DOI:** 10.7759/cureus.88978

**Published:** 2025-07-29

**Authors:** Jessica Cohen, Adi Cohen, Igor Cordeiro de Oliveira, Isabel Jaszewski, Sheela Anasseri, Rebecca Cherner

**Affiliations:** 1 Graduate Medical Education, Nova Southeastern University Dr. Kiran C. Patel College of Osteopathic Medicine, Fort Lauderdale, USA; 2 Family Medicine, Broward Health Medical Center, Fort Lauderdale, USA

**Keywords:** delayed medical care, gangrenous cholecystitis, healthcare literacy, mental health bias, schizophrenia

## Abstract

Schizophrenia is a chronic psychiatric condition that can contribute to delays in the diagnosis and treatment of health conditions. Factors like poor judgment, decreased medical literacy, cognitive barriers, and delayed seeking of therapy can result in worse outcomes of the disease. Acute gangrenous cholecystitis is a severe complication of acute cholecystitis, requiring immediate surgical intervention.

We present the case of a 54-year-old Spanish-speaking male with schizophrenia who presented with seven days of progressively worsening right upper quadrant (RUQ) pain, as well as associated nausea and postprandial emesis. The patient also reported having been recently discharged from an Emergency Department (ED) on an antibiotic regimen after presenting with the same chief complaint. Further evaluation showed laboratory findings of leukocytosis (22.3 × 10³ cells/µL) and a computed tomography (CT) of the abdomen and pelvis with findings consistent with acute cholecystitis, with a possible gangrenous component. This case sheds light on the impact of psychiatric disorders on the treatment of disease. Stigmatized psychiatric comorbidities, including schizophrenia, can lead to worsened outcomes and an increased risk of life-threatening complications due to delayed initiation of proper treatment protocols. Increased awareness, identification of biases, and practiced protocols for disease management should be upheld in patients with mental illness to prevent complications.

## Introduction

Gangrenous cholecystitis is a life-threatening complication of acute cholecystitis, affecting 2% to 20% of patients [1]. Compared to acute cholecystitis, there is a higher mortality rate associated with gangrenous cholecystitis, in the range of 15%-50% [1,2]. Common risk factors include male gender, older age (age > 45 years), delayed surgical management, leukocytosis, diabetes, and underlying cardiovascular disease [2-4]. 

The mechanism of illness originates from cystic duct obstruction, which causes increased wall stress of the gallbladder and subsequent vascular compromise. This can then lead to ischemia with necrosis of the tissue [1]. The clinical presentation of gangrenous cholecystitis is similar to acute cholecystitis, with complaints of severe right upper quadrant (RUQ) pain that may radiate to the back, fever, nausea, and vomiting. Physical exam findings may include arrest of inspiration during deep palpation of the RUQ, known as Murphy’s sign, while labs classically show a high degree of leukocytosis. Some studies have found a white blood cell (WBC) count >15-17 × 10³ cells/µL to be predictive of gangrenous cholecystitis [1,4].

However, due to nonspecific findings on physical exam, clinical diagnosis and prompt intervention for gangrenous cholecystitis can be delayed, especially in marginalized patient populations, such as those with chronic mental illness. This case discusses a patient with a medical history significant for schizophrenia and homelessness, who presents with severe abdominal pain secondary to gangrenous cholecystitis.

## Case presentation

The patient is a 54-year-old Hispanic, homeless male who presented to the Emergency Department (ED) with a seven-day history of progressively worsening RUQ abdominal pain, which radiated to the epigastric region and right flank. The patient also reported associated nausea and postprandial emesis. Per the patient, he was seen three days prior in another acute ED setting but was discharged on an antibiotic regimen. Electronic medical records of this hospital encounter were unavailable. 

The patient’s past medical history includes schizophrenia, polysubstance abuse, and hypertension. Past surgical history is significant for self-inflicted injury to the pinnas of the ears due to persistent auditory hallucinations. On initial evaluation, the patient was noted to be in moderate distress secondary to pain, but alert and oriented to time and place. The abdomen was soft and distended, with no visible lesions or ecchymosis. There was tenderness to palpation in all four abdominal quadrants, but most prominent in the RUQ. Further evaluation revealed a positive Murphy’s sign, with no palpable masses or guarding.

Vital signs on presentation showed an afebrile patient with hypertension (173/105 mmHg). Initial complete blood count (CBC) demonstrated leukocytosis with elevated hemoglobin and hematocrit, glucose, and total bilirubin (Table 1). In the ED, the patient received intravenous (IV) fluids of 1 liter (L) normal saline and 1 L lactated Ringer’s; 4 milligrams (mg) IV ondansetron; 15 mg IV ketorolac; and 4.5 grams (g) IV piperacillin-tazobactam. The Family Medicine team was then consulted for admission.

**Table 1:** Patient lab values collected in the emergency department on initial presentation.

Lab Test	Initial Patient Values	Reference Range
WBC count	22.13 x 10³ cells/µL	4.0 - 11.0 x 10³ cells/µL
Hemoglobin	18.5 g/dL	13.5 - 17.3 g/dL
Hematocrit	53.7%	38.0 - 52.0%
Glucose	126 mg/dL	70 - 105 mg/dL
Total bilirubin	1.3 mg/dL	0.2 - 1.2 mg/dL
Alkaline phosphatase	91 U/L	40 - 150 U/L
Aspartate aminotransferase (AST)	15 U/L	5 - 34 U/L
Alanine aminotransferase (ALT)	< 6 U/L	≤ 55 U/L

Prior to admission, there was debate among the medical providers as to whether psychiatry was needed to determine the patient's capacity for consent to medical intervention, given his history of schizophrenia and continued auditory hallucinations at presentation. The patient was a native Spanish speaker, and the Family Medicine team had a Spanish-speaking provider who assessed the patient's capacity. Specifically, during the patient interview, the team utilized their medical expertise and agreed that the patient met all four criteria of capacity, including the ability to communicate a choice, understand, appreciate, and reason about his medical status and optimal treatment options. Additionally, they consulted the General Surgery team to evaluate the patient, and the Psychiatry team to optimize the patient’s medication regimen.

**Figure 1:**
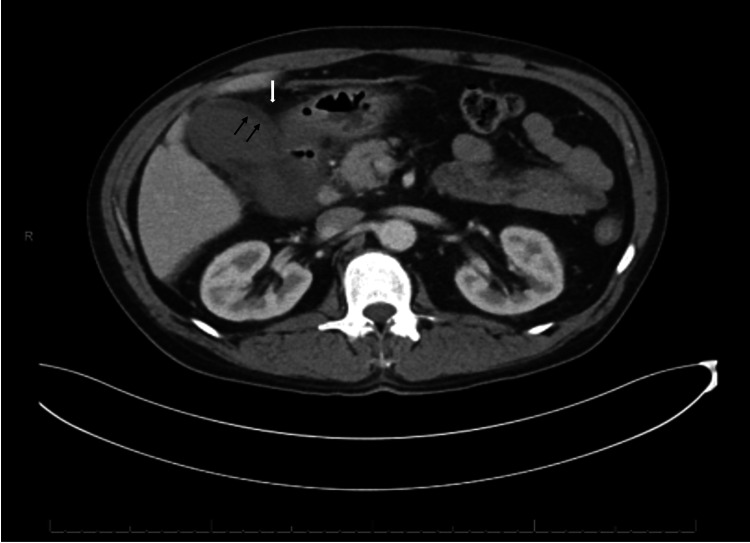
CT of the abdomen and pelvis with contrast, demonstrating significant inflammatory stranding (white arrow), gallbladder wall thickening (black arrows), and an overall loss of enhancement of the gallbladder wall, with associated distention concerning for acute cholecystitis with a possible gangrenous component. CT, computed tomography

**Figure 2:**
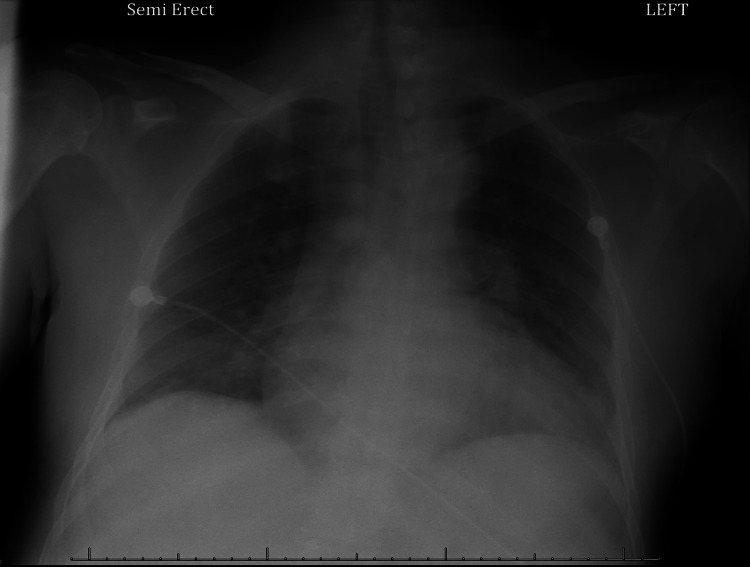
Chest X-ray showing cardiomegaly with mild interstitial pulmonary edema.

Following admission, a computed tomography (CT) scan of the abdomen and pelvis with contrast was performed, which was concerning for acute cholecystitis with possible gangrenous components (Figure 1). No drainable fluid collection was present. A chest X-ray (CXR) was also obtained, and it showed cardiomegaly with mild interstitial pulmonary edema (Figure 2). 

The General Surgery team performed a robot-assisted laparoscopic cholecystectomy for acute gangrenous cholecystitis two days after admission. The patient remained hemodynamically stable throughout the surgery, with no intraoperative complications. Postoperatively, the patient was afebrile, with downtrending leukocytosis and improving liver function tests. Four abdominal ports and a Jackson-Pratt (JP) drain remained clean, dry, and nonerythematous, with the JP drain showing serosanguineous fluid. The patient received multimodal pain management and a medical regimen consisting of 5 mg by mouth (PO) haloperidol, 4.5 g IV piperacillin-tazobactam, 2 mg IV morphine, 100 mg PO thiamine, and 20 mg IV famotidine. The patient was discharged 48 hours postoperatively with removal of the JP drain and medications including 875-125 mg twice daily (BID) amoxicillin-clavulanate for seven days, 3.125 mg carvedilol, 80 mg daily valsartan, and 100 mg daily thiamine. Outpatient follow-up was recommended in one to two weeks.

## Discussion

Those with delayed surgical intervention for acute cholecystitis are at increased risk of developing complications, such as gangrenous cholecystitis [1]. Clinical findings supporting the patient’s diagnosis of gangrenous cholecystitis include severe RUQ pain, positive Murphy’s sign, WBC count >17 × 10³ cells/µL, as well as abdominal CT findings showing overall loss of enhancement of the gallbladder wall [1,5]. 

Per the patient’s verbal record, he was seen just a few days prior in an ED due to severe RUQ abdominal pain and was discharged with antibiotics. Of note, we were unable to obtain written records from this patient's prior encounter and have limited knowledge of the actual course of, and/or intended, patient care. However, the standard of care for patients with acute cholecystitis is early laparoscopic cholecystectomy, and it has been shown that patients treated only with antibiotics experience a recurrence of symptoms in 2.5%-22% of cases [6]. Additionally, further delay of treatment for gangrenous cholecystitis can lead to life-threatening complications, such as gallbladder perforation, infection, and sepsis [3].

The conservative treatment route is also suboptimal, given the patient's socioeconomic status. A cohort study comparing homeless and non-homeless individuals following discharge from a psychiatric hospital in Ontario, Canada, found that 46.3% of the homeless patients had no medical care in the 30 days following hospital discharge [7]. It was also found that, prior to admission, homeless individuals - compared to their housed counterparts - were less likely to be seen by a primary care physician and more likely to have had hospitalizations for both mental and non-mental health reasons [7]. 

Barriers preventing homeless individuals from adequate post-discharge follow-up include a lack of identifying documentation and insurance, costs of medical care, biases, lack of transportation, and difficulty making and receiving appointments [7,8]. Thus, these limitations faced by the patient proved conservative treatment to be the less practical option. 

Next, it was discussed whether to consult a psychiatrist to determine if the patient had the capacity to consent to surgical intervention. Capacity is a clinical assessment of patients' abilities to make their own decisions and has four components: the ability to communicate a choice, understanding, appreciation, and rationalization/reasoning [9]. Psychiatrists can be consulted to determine capacity in complex patient cases and in situations that are not emergent. However, for patients in acute settings, any licensed physician should be able to determine capacity [10]. In this case, with a clinical picture of gangrenous cholecystitis, the Family Medicine team was able to determine the patient's capacity, which helped him receive the surgical intervention needed for optimal care. 

Unfortunately, schizophrenic patients are highly stigmatized among healthcare professionals [11]. It is critical to continue dismantling these conscious and unconscious biases among healthcare professionals and to ensure the adequate delivery of proper care for these marginalized individuals. 

## Conclusions

This case evaluates the challenges in diagnosing and managing gangrenous cholecystitis in a patient with schizophrenia and homelessness. Barriers to healthcare access, delayed seeking of care, limited resources, and bias among healthcare workers led to a delay in surgical intervention, ultimately leading to the complication of gangrenous cholecystitis in this case. Doctors must maintain and uphold appropriate clinical standards when evaluating all patients with a chronic medical health condition, especially those in acute and emergent situations. This case suggests that a multidisciplinary approach, including an efficient capacity assessment, surgical consultation, and improved communication, is important to prevent delays in care and boost patient outcomes.
